# Mitochondrial genome polymorphism in the East Slavic population of Northeastern Siberia

**DOI:** 10.18699/vjgb-25-77

**Published:** 2025-09

**Authors:** B.A. Malyarchuk, G.A. Denisova, A.N. Litvinov

**Affiliations:** Institute of Biological Problems of the North of the Far Eastern Branch of the Russian Academy of Sciences, Magadan, Russia; Institute of Biological Problems of the North of the Far Eastern Branch of the Russian Academy of Sciences, Magadan, Russia; Institute of Biological Problems of the North of the Far Eastern Branch of the Russian Academy of Sciences, Magadan, Russia

**Keywords:** mitochondrial genome, genetic polymorphism, molecular phylogeography, Eastern Slavs, Northeastern Siberia, митохондриальный геном, генетический полиморфизм, молекулярная филогеография, восточные славяне, Северо-Восток Сибири

## Abstract

Data on mitochondrial DNA (mtDNA) polymorphism at the population level are of significant interest to researchers in the fields of population and ethnic genetics, forensic medicine, and forensic science. In the present study, we have obtained data on the variability of whole mitochondrial genomes in the immigrant East Slavic population of Northeastern Siberia (using the Magadan region as an example). The study yielded novel data concerning mtDNA variability in the Magadan region’s inhabitants comprising maternal lineages of Russians (N = 49) and Ukrainians (N = 15), as well as individuals with a mixture of maternal and paternal ancestries, including Russians on the maternal side and indigenous populations (Koryaks, Evenes, and Itelmens) on the paternal side (N = 4). In addition, the mitogenomes of the Russian population from the Novgorod, Kaluga, and Yaroslavl regions (N = 15) were sequenced to enhance the power of the phylogeographic analysis. The results of the study demonstrated that the mitochondrial gene pool of the East Slavic immigrant population in the Magadan region is characterized by a high level of diversity. The analysis of genetic differentiation of Russian populations within Russia, as measured by the variability of complete mitochondrial genomes, revealed a low level of interpopulation differences (Fst = 0.15 %, P = 0.2). The results of multidimensional scaling of Fst distances indicate that the Russians residing in the Magadan region are genetically similar to the Russian populations inhabiting the southwestern part of the country, specifically the Belgorod and Orel regions. The gene pool of the Russian population in the Magadan region is predominantly characterized by mtDNA haplotypes of West Eurasian (including European) origin. The prevalence of East Asian-derived haplotypes among the Russian population is relatively low, accounting for approximately 4.8 % of the total. However, certain East Asian-specific haplogroups, such as F1b1 and Z1a1a, have demonstrated a prolonged presence in the gene pools of Eastern European populations, as evidenced by phylogeographic analysis. Among the European mtDNA haplotypes of Russians from the Magadan region, Eastern European variants predominate, and they also have a high proportion of mtDNA haplotypes specific to Slavs (19.4 %). Furthermore, rare mtDNA haplotypes have been identified in the mitochondrial gene pools of Russians and Ukrainians residing in the Magadan region. These rare haplotypes are linked to the maternal lines of Empress Alexandra Fedorovna Romanova (haplogroup H1af2) and Prince Dmitry, son of Prince Alexander Nevsky (haplogroup F1b1-a3a2a).

## Abstract (ru)

Информация о полиморфизме митохондриальной ДНК (мтДНК) на популяционном уровне вызывает большой интерес со стороны исследователей в области популяционной и этнической генетики, cудебной медицины и криминалистики. В настоящей работе получены данные об изменчивости целых митохондриальных геномов у пришлого восточнославянского населения Северо-Востока Сибири (на примере Магаданской области), а также новые сведения об изменчивости мтДНК у жителей Магаданской области – русских (N = 49) и украинцев (N = 15) – по материнской линии, а также метисного происхождения – русских по материнской линии и коренных жителей (коряки, эвены, ительмены) по отцовской линии (N = 4). Кроме этого, для повышения информативности филогеографического анализа секвенированы митогеномы русских Новгородской, Калужской и Ярославской областей (N = 15). Результаты проведенного исследования показали, что митохондриальный генофонд пришлого восточнославянского населения Магаданской области характеризуется высоким уровнем генетического разнообразия. Результаты анализа генетической дифференциации популяций русского населения России по данным об изменчивости целых митохондриальных геномов свидетельствуют о низком уровне межпопуляционных различий (Fst = 0.15 %, P = 0.2). По результатам многомерного шкалирования Fst-дистанций русские Магаданской области кластеризуются с русскими юго-западной части страны – популяциями Белгородской и Орловской областей. В генофонде русского населения Магаданской области преобладают гаплотипы мтДНК западноевразийского (включая европейское) происхождения. Доля гаплотипов восточноазиатского происхождения у русских невелика (4.8 %), однако часть из них (гаплогруппы F1b1 и Z1a1a), согласно результатам филогеографического анализа, длительное время эволюционировала в генофондах популяций Восточной Европы. Среди европейских по происхождению гаплотипов мтДНК у магаданских русских преобладают восточноевропейские варианты, а также у них высока доля гаплотипов мтДНК, специфичных для славян (19.4 %). В митохондриальных генофондах русских и украинцев Магаданской области обнаружены редкие гаплотипы, родственные материнским линиям императрицы Александры Федоровны Романовой (гаплогруппа H1af2) и князя Дмитрия, сына князя Александра Невского (гаплогруппа F1b1-a3a2a).

## Introduction

Mitochondrial DNA (mtDNA) is a maternally inherited, nonrecombining genetic system that is highly informative for studying the genetic history of populations and reconstructing migrations. The utilization of mtDNA markers to investigate the emergence processes of Siberian immigrant populations of Eastern European (predominantly Russian) origin dates back to the initial population studies of mitochondrial DNA polymorphism in Russia (Lemza, Sokolova, 1992; Malyarchuk et al., 1994; Derenko, Malyarchuk, 1996; Kazakovtseva et al., 1998). Recent studies have demonstrated that the migrant population of Siberia exhibits a high diversity of mitochondrial lineages, predominantly of European origin (Gubina et al., 2014). This phenomenon can be attributed to historical migration processes that commenced in the 16th–17th centuries, coinciding with the exploration of Siberia by Russian pioneers. These processes persisted throughout the capitalist and socialist development of Russian society, contributing to the contemporary genetic landscape of the region.

Meanwhile, investigations of mtDNA polymorphism in the Russian Old Believers of Siberia, who diverged in the mid- 17th century, demonstrated that the genetic composition of the Old Believers aligns with that of European populations, including Russians. Nevertheless, the prevalence of the East Asian component in the gene pool of the Russian Old Believers is marginally higher compared to contemporary Russians residing in the Novosibirsk Oblast (Gubina et al., 2014). This observation may be attributed to the increased level of intermarriage between the Old Believers and indigenous Siberians, living in close proximity for several centuries.Meanwhile, investigations of mtDNA polymorphism in the Russian Old Settlers – descendants of Russian servants and traders who established themselves in the Far North of Eastern Siberia from the mid-17th century onward – revealed a remarkably elevated proportion of East Asian genetic patterns in the gene pools of the Russian Old Settlers (100 % in Pokhodsk and Markovo residents, 67 % in Russkoe-Ust’e residents) (Sukernik et al., 2010; Borisova et al., 2024). This phenomenon can be attributed to the unique historical development of the Russian Old Settlers’ populations, characterized by intermarriage between immigrants from Eastern Europe (Cossacks, merchants, and industrialists) and indigenous women, predominantly of Yukaghir origin (Sukernik et al., 2010; Solovyev et al., 2023; Borisova et al., 2024).

The genetic structures of modern indigenous populations in the northeast of Siberia are similarly organized. The gene pools of the Koryaks, Evens, and Chukchi also show asymmetry in the contribution of maternal (by mtDNA) and paternal (by Y chromosome) lineages of European origin, with paternal lineages of this kind sharply prevailing over maternal lineages (Balanovska et al., 2020; Agdzhoyan et al., 2021; Derenko et al., 2023; Solovyev et al., 2023; Borisova et al., 2024; Malyarchuk, Derenko, 2024). The initial settlements established by Russian explorers and traders (Tauysk, Gizhiginsk, Yamsk, Ola, and other settlements) emerged in the 17th–18th centuries within the boundaries of Magadan Oblast. However, it was not until the 1930s that a significant influx of individuals, numbering in the hundreds of thousands, arrived in the region, primarily driven by the economic development of this prosperous Siberian territory. Consequently, the Magadan Oblast began to form the so-called newcomer population, which was predominantly comprised of Russians (81.5 %) and Ukrainians (6.3 %) (according to Rosstat data from 2010). The indigenous aboriginal population constituted approximately 3 % of the total Magadan Oblast population, numbering 133,387 individuals (according to Rosstat data from 2024).

In this paper, we present data on the variability of whole mitochondrial genomes in the immigrant population of the Magadan region. Our objective is to use these data to identify phylogeographic patterns of mitochondrial haplotypes and to study interpopulation relationships of the Russian population of the Russian Federation

## Materials and methods

Mitochondrial genome sequences of the Russian residents of the Magadan region (Magadan city and settlements of the Severo-Evensky district; N = 49) were determined. The mitochondrial genomes of Magadan residents were also subjected to phylogeographic analysis, which included 15 Ukrainians (on the maternal side) and four individuals of mixed ancestry, comprising Russians on the maternal side and indigenous peoples (Koryaks, Evenes, and Itelmens) on the paternal side (Table S1 in the Supplementary Materials)1.


Supplementary Materials are available in the online version of the paper: https://vavilov.elpub.ru/jour/manager/files/Suppl_Malyarchuk_Engl_29_5.xlsx


Furthermore, the mitogenomes of the Russians from the Novgorod, Kaluga, and Yaroslavl oblasts (N = 10, 3, and 2, respectively) were sequenced to enhance the comprehensiveness of phylogeographic analysis (Table S1). The methods and approaches employed for sequencing whole mtDNA molecules were previously described (Derenko, Malyarchuk, 2010). The nucleotide sequences of mitochondrial genomes were deposited in GenBank (www.ncbi.nlm.nih.gov) under accession numbers PQ285752-PQ285800, PQ300111-PQ300129, and PQ283331-PQ283345.

Phylogenetic relationships between mtDNA nucleotide sequences were analyzed using the maximum parsimony method implemented in the mtPhyl v4.015 program (https:// isogg.org/wiki/MtPhyl). During the construction of the tree, length polymorphism at sites 16180-16193, 309-315, and 514-524 as well as substitution at position 16519 were not considered. To determine the evolutionary age of monophyletic mtDNA clusters, we employed the molecular clock incorporated in the mtPhyl program based on the correction of the long-term phylogenetic rate of mutations in human mtDNA (one nucleotide substitution in the whole mitogenome over 3,624 years, or 1.665 × 10–8 substitutions per site per year), taking into account the effect of negative selection (Soares et al., 2009).

The phylogenetic classification of mtDNA haplogroups proposed by the developers of the PhyloTree resource (www. phylotree.org) was utilized as a foundation for this study. Furthermore, the regularly updated classification of mtDNA variants provided by the YFull MTree resource (https://www. yfull.com/mtree/) was also considered. The designation of monophyletic mtDNA clusters as ethnospecific was made on the following criteria: at least 75 % of mtDNA haplotypes in the cluster were found to be characteristic of representatives of a certain ethnic group (e. g., Russians) or ethnic community (Slavs). For the phylogeographic analysis of mtDNA, we utilized data on the variability of complete mitogenomes in individuals representing diverse human populations, as reported in GenBank, the Logan DNA Project (http://www. ianlogan.co.uk), and YFull MTree. At the end of 2024, the GenBank database has accumulated a collection of over 61,000 mitochondrial genomes, representing a diverse array of ethnic groups from various geographical regions worldwide (https://www.mitomap.org/foswiki/bin/view/MITOMAP/ GBFreqInfo). The ethnic affiliation of the studied samples was determined through the utilization of information derived from the available databases

In order to undertake a comparative analysis of complete mtDNA variability at the population level, we employed previously published data for the Russian populations of the European part of the country (Malyarchuk et al., 2017). In addition to 49 new mtDNA sequences, the Russian samples from the Magadan region included 13 previously sequenced mitochondrial genomes (Table S1). The genetic diversity parameters of populations were calculated using the DnaSP 5.10.01 software package (Librado, Rozas, 2009). The analysis of molecular variability (AMOVA, Fst-analysis) based on pairwise nucleotide differences between mitogenomes was performed using the Arlequin 3.5.1.2 software package (Excoffier, Lischer, 2010). The location of populations in two-dimensional space was investigated using the multidimensional scaling method of interpopulation Fst-differences, implemented in the STATISTICA10 software package (StatSoft Inc.).

## Results and discussion

**Interpopulation differences of the Russian population based on whole mitochondrial genome variability data**

An analysis of mtDNA variability revealed that the studied Russian samples from the Magadan region (49 new and 13 previously sequenced mitogenomes) do not show significant differences at the population level in the primary parameters of genetic diversity in comparison with other Russian populations of the European part of Russia (Table 1). According to these results, the Magadan Russians occupy an intermediate position among the Russian populations, where the lowest values of the average number of pairwise nucleotide differences (k) are observed among the Russians of the Pskov and Novgorod regions, and the highest, among the Russians of the Vladimir region. Tajima’s D parameter, which tests the neutrality of mtDNA evolution within populations, has a significantly negative value in the Magadan region, a finding also observed in other European populations, including Russians (Litvinov et al., 2020). This suggests the influence of negative selection on mtDNA variability.

**Table 1.**
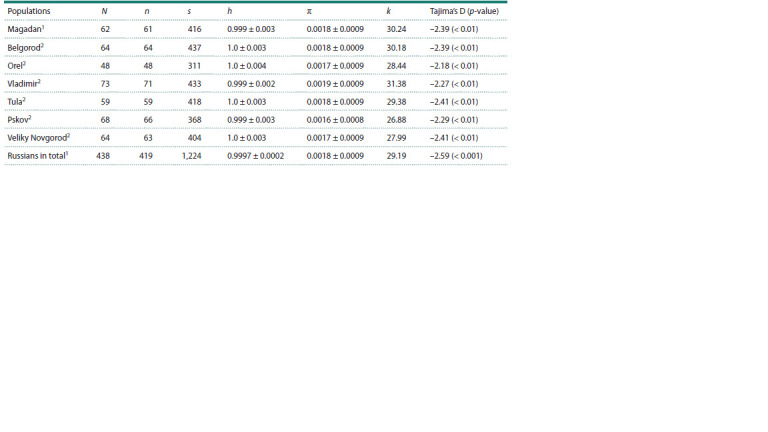
Genetic parameters in Russian populations according to the data on variability of whole mitochondrial genomes Notе. N – sample size; n – number of haplotypes; s – number of polymorphic sites; h – haplotypic diversity; π – nucleotide diversity; k – average number of pairwise nucleotide differences. References: 1 present study; 2 Malyarchuk et al., 2017.

**Fig. 1.**
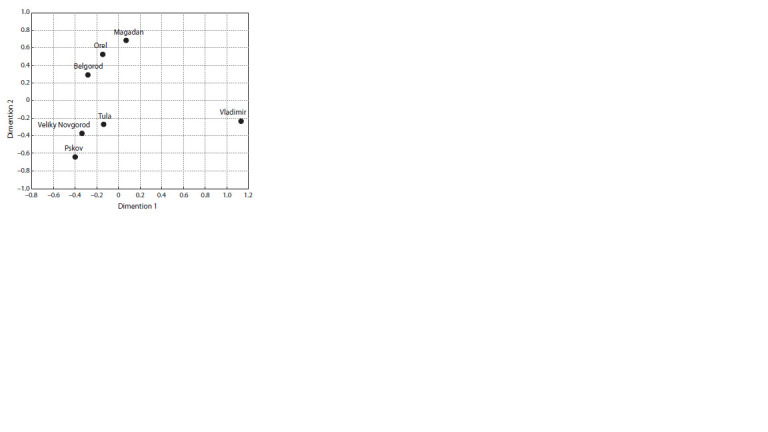
Results of multidimensional scaling of interpopulation Fst-values based on pairwise nucleotide differences between sequences of whole mitochondrial genomes from different Russian populations. The stress value is equal to 0.00003.

The analysis of Fst-differences between mitochondrial genomes in Russian populations showed the absence of interpopulation differences (Fst = 0.15 %, P = 0.2). Pairwise comparisons revealed statistically significant differences between the population of the Vladimir region and the northwestern Russian populations of the Pskov and Novgorod regions (Table 2). Conversely, the results of multidimensional scaling of interpopulation Fst-differences indicate that the Russian population of the Magadan region is grouped with the southwestern populations of the Belgorod and Orel regions (Fig. 1). This grouping is significantly different from the Russians of the Vladimir region, as can be seen from the results of multidimensional scaling.

**Table 2.**
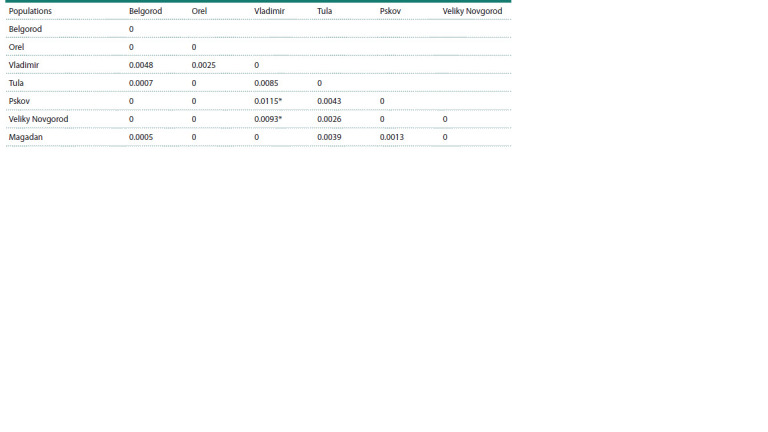
Fst-differences between Russian populations according to the data on the variability of nucleotide sequences of whole mitogenomes Notе. Fst-values are based on pairwise nucleotide differences between mtDNA haplotypes and are shown under the diagonal. * Statistically significant differences (P <0.05).

**Phylogeographic analysis of mtDNA haplotypes of the East Slavic immigrant population of the Magadan region**

The gene pool of Russian population of Magadan region is represented mainly by Western Eurasian mtDNA haplogroups (95.2 %) (Table S1). The most prevalent are haplogroups H (37.1 %), T (16.1 %), U (14.5 %), J (6.5 %), and HV (4.8 %). Other Russian populations in Eastern Europe have similar mitochondrial haplogroup spectra (Morozova et al., 2012; Kushniarevich et al., 2015; Malyarchuk et al., 2017).

The results of the phylogenetic analysis revealed the presence of 62 different mtDNA haplotypes within the cohort of the Russian population residing in the Magadan region (N = 62). This indicates that the sample did not contain identical haplotypes. Furthermore, phylogeographic analysis of the mtDNA haplotypes identified in the Magadan Russians revealed that these genetic variants are predominantly distributed within the European population (refer to Table S1 and Figure S1 for more details). A total of three distinct haplotypes were identified as belonging to haplogroups that are prevalent in West Asia, including H-7630-11113-12172, R0a1a5, and M5a1b, with samples 10_R, 2_R, and 44_R corresponding to these haplogroups, respectively. However, the origins of two additional haplotypes could not be determined. Approximately a quarter of the Magadan sample (25.8 %) consisted of mtDNA haplotypes common in Eastern European populations, with almost 20 % of the Magadan Russian haplotypes belonging to mtDNA subgroups predominantly distributed among Slavs (haplogroups HV-15617, HV6a1, H1b2g, H13a2b, U4d2b, U5a1a1h, U5a2b1g, U5a2a1o, K1c1e, K2b1, J1c3a1, V7a) (Fig. S1). A proportion of the samples (11.3 %) were found to belong to haplogroups that had been identified primarily within the Russian population (H5a1a, J1c4b1, I1a1c-10454, W1c-10086-12136, R1a1a1, F1b1-a3a2a). The frequency of the Baltic-Finnish component (H1n4, H49, U5b1b1a, Z1a1a) was 8.1 %.

**Fig. 2.**
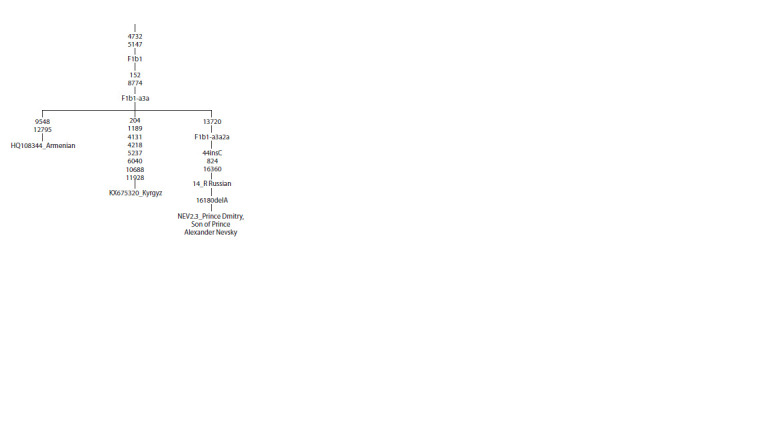
Phylogenetic tree of mtDNA of haplogroup F1b1-a3a. Nucleotide positions at which transitions occurred are shown on the branches; 16180delA indicates deletion A at position 16180; the GenBank sample numbers and ethnicity are also indicated

Three mtDNA haplotypes identified in the Russians of the Magadan region belong to haplogroups of East Asian origin: F1b1, Z1a1a, and N9a2a2. However, the phylogeographic analysis demonstrated that haplogroup N9a2a2 is exclusively distributed in East Asia, as it was detected only in the Japanese population (Fig. S1). The presence of the Z1a1a haplotype suggests a potential contribution of Finno-Ugric tribes to the ethnic history of Russians (Lunkina et al., 2004). This mtDNA haplogroup is prevalent in populations from Northeastern Europe, particularly in the Fennoscandian region. It is hypothesized that this haplogroup was introduced into the gene pool of the Sami and Finns from the Volga-Urals region about 3,000 years ago (Tambets et al., 2004; Ingman, Gyllensten, 2007). The F1b1-haplotype identified in the Magadan Russians is noteworthy for its association with the F1b1-a3a2a subgroup, which, according to the YFull MTree classification system, is predominantly present in the modern Russian populations of the Kursk, Belgorod, and Bryansk regions (Table S1). Phylogenetic analysis showed that this haplotype is almost identical (except for deletion at position 16180) to the F1b1-haplotype identified in Prince Dmitry, son of Prince Alexander Nevsky, and possibly of Cuman origin (Zhur et al., 2023) (Fig. 2).

**Fig. 3.**
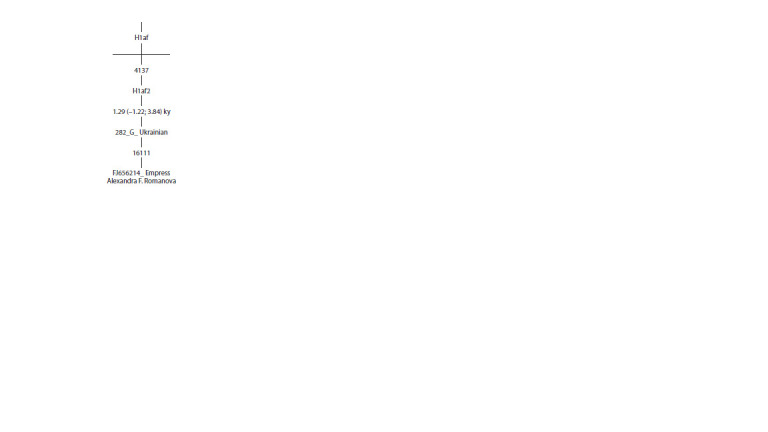
Phylogenetic tree of mtDNA of haplogroup H1af. Nucleotide positions at which transitions occurred are shown on the branches, and the evolutionary age of the H1af2 subgroup in thousands of years (ky) is also indicated

Phylogeographic analysis of 15 mitogenomes of Ukrainian origin also demonstrated that the identified mtDNA haplotypes are predominantly distributed in European populations (Table S1). Three of these haplotypes belong to Slavic-specific mtDNA haplogroups (H6c1, H1u5a2, HV9b1a1), while one more belongs to haplogroup U5b1b1a1d, which is predominantly distributed among Finns. Notably, the Ukrainian sample from the Magadan region exhibited a rare haplotype belonging to haplogroup H1af2, which is known to be associated with the maternal lineage of Empress Alexandra Fedorovna Romanova (Fig. 3).

Despite the substantial size of the GenBank database, which includes more than 61,000 mitogenomes from diverse ethnic backgrounds, it lacks other H1af2 sequences. In contrast, the YFull MTree database contains additional H1af2 sequences (YF129594 and YF109892). However, both databases lack information on ethnic origin and the nucleotide sequences themselves. Our study enabled the detection of an H1af2 haplotype associated with the Russian Tsarina in an individual of Ukrainian origin. The evolutionary age of the H1af2 subgroup was determined to be about 1.3 thousand years, and the YFull MTree database suggests a slightly higher estimate of 3.2 thousand years. Genealogical data indicate that the maternal lineage of the Russian Empress (or Queen Victoria of Great Britain, respectively) is of Western European (especially French) originPhylogeographic analysis of individuals of mixed ancestry revealed that the haplotypes of two individuals (Koryak on the paternal side) belong to haplogroups found predominantly among Finns (K1c1 and U5b1b1a). The remaining two mtDNA haplotypes are associated with haplogroups for which the precise geographical origins remain uncertain; both J2b1a11 and U5a1a1a are common in European populations

## Conclusion

The results of the study demonstrated that the mitochondrial gene pool of the East Slavic immigrant population in Northeastern Siberia (with a focus on the Magadan region) exhibited a high level of diversity. At the same time, the genetic differentiation of Russian populations, as determined by the analysis of variability in whole mitochondrial genomes, was found to be remarkably low. This finding suggests a high degree of similarity among mtDNA haplotypes in diverse Russian populations. The mitochondrial gene pool of the Russian population of the Magadan region does not differ significantly from the gene pools of other Russian populations of the European part of the country. According to the results of multidimensional scaling of Fst-distances, the Magadan Russians cluster with the Russian populations of the southwestern part of the country, specifically the Belgorod and Orel regions. This result indicates a distinctive characteristic of the contemporary population of Northeastern Siberia in comparison with the populations of the Russian Old Settlers, who established themselves in the Far North of Eastern Siberia since the middle of the 17th century. The Russian Old Settlers have a markedly high prevalence of East Asian (predominantly Yukaghir) mtDNA haplotypes (Sukernik et al., 2010; Borisova et al., 2024). The analysis of Y chromosome polymorphism and autosomal loci shows that the European variants of polymorphism preserved in these populations (along with the Russian language and culture) are predominantly associated with the population of Northeastern Europe. This finding lends support to the “Pomor” hypothesis of the origin of the Russian Old Settlers of the Arctic coast (Solovyev et al., 2023).

An analysis of the gene pool of the Russian and Ukrainian inhabitants of the Magadan region shows that the mtDNA haplotypes of European origin are predominant. The proportion of East Asian mtDNA haplotypes among Russians is minimal (4.8 %), and further analysis using phylogeographic methods revealed that F1b1 and Z1a1a haplotypes, despite their East Asian origin, have undergone extensive evolution within the gene pools of Eastern European populations. Among mtDNA haplotypes of European origin, Eastern European variants predominate in the Magadan region Russians, accounting for a significant proportion (19.4 %) of mtDNA haplotypes specific to Slavs. Such high frequencies of Slavic-specific mtDNA lineages have been previously reported only among Ukrainians – 23.6 % (Malyarchuk, Derenko, 2023).

It is interesting to note that the findings in the mitochondrial gene pools of modern Russians and Ukrainians have revealed the presence of rare haplotypes that appear to be related to the maternal ancestry of Empress Alexandra Fedorovna Romanova and Prince Dmitry, son of Prince Alexander Nevsky. These findings may offer a promising avenue for further research.

## Conflict of interest

The authors declare no conflict of interest.
